# Regarding: ‘The Role of EphrinB2–EphB4 Signalling Pathway in Regeneration of Inflammatory Bone Defect’

**DOI:** 10.1111/jcmm.71031

**Published:** 2026-01-16

**Authors:** Yue Feng, Yantao Zhao

**Affiliations:** ^1^ First Operating Room Central Hospital of Dalian University of Technology Dalian China; ^2^ Department of Joint Surgery Central Hospital of Dalian University of Technology Dalian China

To the Editor,

The recent study by Shen et al., ‘The Role of EphrinB2–EphB4 Signalling Pathway in Regeneration of Inflammatory Bone Defect’, provides valuable experimental evidence linking the EphrinB2–EphB4 axis to bone regeneration under inflammatory conditions [1]. Using a TNF‐α–induced mandibular defect model, the authors elegantly demonstrated that suppression of EphB4 signalling attenuates osteogenic differentiation while promoting osteoclast activity, resulting in impaired bone repair. This work highlights the central role of bidirectional Eph–ephrin communication in maintaining the delicate balance between osteoblast and osteoclast function essential for skeletal homeostasis [2, 3].

The study is technically robust, integrating molecular assays with histological and immunohistochemical analyses. However, several aspects merit deeper consideration. One limitation is the lack of mechanistic interrogation of downstream pathways. EphB4 is known to interact with intracellular cascades such as Wnt/β‐catenin, ERK/MAPK, and PI3K/Akt, which are strongly influenced by TNF‐α and play vital roles in osteoblast differentiation and survival [4, 5]. Clarifying whether the observed suppression of osteogenesis in EphB4‐deficient mice is mediated via these canonical pathways or through cross‐talk with the RANKL/NF‐κB system would enhance mechanistic depth and biological coherence.

In addition, the protein‐level validation is relatively limited. Only Runx2 and BSP were examined, which provide a partial picture of osteoblast maturation. Including additional markers—such as COL1A1, OPN, and ATF4—and key osteoclastogenic proteins like NFATc1 or CTSK could yield a more complete overview of bidirectional differentiation [6]. Advanced imaging modalities, including micro‐CT‐based morphometry or calcein double labeling, would also permit quantitative assessment of bone formation beyond histological description [7].

Another interesting observation is that EphrinB2 knockdown produced minimal effects on bone regeneration, which contrasts with earlier reports that EphrinB2 overexpression enhances osteogenic potential in mesenchymal and dental pulp stem cells [8, 9]. This inconsistency may stem from functional redundancy within the Ephrin family. EphrinB1 shares considerable structural homology with EphrinB2 and can mediate reverse signalling via alternative EphB receptors (EphB2/B3) [10]. Employing dual‐gene knockdown or conditional knockout models would clarify the specific contributions of these ligands in vivo and strengthen the conclusion.

From a translational standpoint, the study's implications are noteworthy. Pharmacologic activation of EphB4 signalling may represent a promising approach for enhancing bone regeneration in chronic inflammatory conditions such as periodontitis or peri‐implantitis. Preclinical evidence suggests that EphrinB2‐Fc fusion proteins or EphB4 agonists can promote osteoblast differentiation while suppressing osteoclastogenesis [11]. Integrating these therapeutic strategies into inflammatory defect models could transform the findings from descriptive biology into clinically actionable insights.

In conclusion, Shen et al. have convincingly shown that EphB4 signalling is indispensable for effective bone regeneration under inflammatory stress, acting as both a promoter of osteogenesis and a suppressor of excessive bone resorption. Future studies incorporating downstream signalling validation, compensatory receptor analysis, and targeted therapeutic activation may further elucidate the translational potential of the EphrinB2–EphB4 axis in managing inflammatory bone loss. This work represents a meaningful step toward understanding how molecular signalling pathways can be leveraged for regenerative therapy.

## Author Contributions

**Yue Feng:** investigation, writing – original draft. **Yantao Zhao:** conceptualization, investigation, writing – original draft, writing – review and editing.

## Ethics Statement

The authors have nothing to report.

## Consent

The authors have nothing to report.

## Conflicts of Interest

The authors declare no conflicts of interest.

## Data Availability

The authors have nothing to report.
